# Promising outcomes of a national programme for the prevention of mother-to-child HIV transmission in Addis Ababa: a retrospective study

**DOI:** 10.1186/1472-6963-10-267

**Published:** 2010-09-09

**Authors:** Alemnesh H Mirkuzie, Sven Gudmund Hinderaker, Odd Mørkve

**Affiliations:** 1 Centre for International Health, University of Bergen, Overlege Danielssens Hus, Årstav. 21, Bergen 5020, Norway; 2 College of Medical and Health Sciences, Department of Nursing and Midwifry, Hawassa University, Awassa, p.o.box 1560, Ethiopia

## Abstract

**Background:**

Prevention of Mother-to-Child HIV Transmission (PMTCT) is still the most effective intervention in combating new HIV infections. In 2008, revised national PMTCT guidelines that incorporated new policies on HIV counselling and testing, antiretroviral prophylaxis regimen and infant HIV diagnosis came into effect in Ethiopia. In the present study we have examined trends in PMTCT service utilization and assessed the rate of MTCT in relation to policy changes in the national PMTCT programme.

**Methods:**

Reports from February 2004 to August 2009 were reviewed in 10 sub-cities in Addis Ababa, Ethiopia. The data was collected from May to October 2009.

**Results:**

The proportion of women who received HIV counselling and testing among new antenatal care attendees increased from 50.7% (95% CI 50.2-51.2) in 2007 to 84.5% (95% CI 84.1-84.9) in 2009 following the shift to routine opt-out testing. Nevertheless, in 2009 only 53.7% of the positive women and 40.7% of their infants received antiretroviral prophylaxis. The HIV prevalence among antenatal attendees decreased significantly from 10.5% in 2004 to 4.6% in 2009 in parallel to the increased number of women being tested. The HIV positive women were over 18 times (RR 18.5, p < 0.0001) more likely to be referred for treatment, care and support in 2009 than in 2004. The proportion of partners tested for HIV decreased by 14% in 2009 compared to 2004, although the absolute number was increasing year by year. Only 10.6% (95% CI 9.9-11.2) of the HIV positive women completed their follow up to infant HIV testing. The cumulative probability of HIV infection among babies on single dose nevirapine regimen who were tested at >=18 months was 15.0% (95% CI 9.8-22.1) in 2007, whereas it was 8.2% (95% CI 5.55-11.97) among babies on Zidovudine regimen who were tested at >=45 days in 2009.

**Conclusion:**

The paper demonstrates trends in PMTCT service utilization in relation to changing policy. There is marked improvement in HIV counselling and testing service utilization, especially after the policy shift to routine opt-out testing. However, despite policy changes, the ARV prophylaxis uptake, the loss to follow up and the partner testing have remained unchanged across the years. This should be a matter of immediate concern and a topic for further research.

## Background

Prevention of mother-to-child HIV transmission (PMTCT) is still the most effective intervention in combating new HIV infections [1]. When the possibility of having an efficacious vaccine seems questionable, as reported in a recent vaccine trial, holding on to PMTCT programmes gives some hope [2]. PMTCT is a multifaceted intervention. It is not just a way to stop vertical transmission of HIV but also to provide access to treatment, care and support for women who would otherwise not get the chance to know their HIV status before it is too late [1].

Despite its importance, a PMTCT programme often suffers poor resource allocation that could threaten programme success [3]. Globally, the challenges in PMTCT programme implementation combined with ever changing scientific advances call for frequent revisits of policies and strategies. In Ethiopia, the first PMTCT guidelines were developed in 2001, incorporating early recommendations by the WHO on HIV counselling and testing, ARV prophylaxis regimen, infant feeding counselling, infant HIV diagnosis algorithm, partner testing and referring HIV positive pregnant women for treatment, care and support [4]. During the recent years, the WHO has made several policy changes to improve PMTCT programme performance. In 2004, the HIV counselling and testing policy was shifted from client initiated opt-in approach to routine opt-out approach in order to improve women's access to prevention interventions and to contain HIV testing within the standard of care for pregnant women [5]. In 2006, the antiretroviral (ARV) prophylaxis regimen was changed from short course single dose NVP (sdNVP) to a more efficacious multidrug zidovudine (ZDV) regimen [1,6]. The infant feeding recommendation was also revised in 2006, when exclusive breast feeding became the preferred method for the first six months, plus complementary feeding from six months. As alternative feeding method, exclusive formula was recommended if formula was acceptable, feasible, affordable, sustainable and safe (AFASS) [7].

The national PMTCT guidelines were revised in 2007, incorporating the policy changes made by the WHO from 2004 to 2006, while retaining the early recommendations on partner testing [6]. The revised guidelines state that all pregnant women undergoing HIV counselling and testing should be advised to bring their partner for HIV testing [6]. Partner testing and involvement in PMTCT is intended to facilitate the women's coping with their test results, adherence to PMTCT recommendations and to facilitate disclosure [8]. However, the potentials of partner testing in PMTCT settings for the prevention of horizontal transmission of HIV is often neglected. Orne-Gliemann et al. described that the large proportion of new HIV infections is occurring in conjugal relationship [9]. Studies from southern and eastern Africa also reported that the prevalence of HIV sero-discordance is within the range of 36%-85% [3,10]. Pregnant women who were HIV negative in early pregnancy could be at risk of acquiring new infection if they had a positive partner at home, which in turn would increase the risk of mother-to-child HIV transmission (MTCT).

In Ethiopia a Monitoring and Evaluation system for HIV/AIDS (M&E) was first launched in 2003 [11]. The system was established to support and strengthen evidence based performance monitoring for HIV/AIDS related interventions [4]. As part of the M&E system, monthly PMTCT reports have been collected from different service outlets to evaluate the performance of the national PMTCT programme at facility, sub-city and region level since the launching of the programme. However, despite the availability of comprehensive PMTCT reports the impact of the programme has not been documented. In this study we make use of the available monthly PMTCT reports from the launching of the PMTCT programme in 2004 to August 2009 in order to examine trends in PMTCT service utilization and to assess the rate of MTCT in relation to policy changes.

## Methods

A retrospective study was conducted from May to October 2009. PMTCT monthly reports from February 2004 to August 2009 were reviewed in all the 10 sub-cities of Addis Ababa. The population of Addis Ababa is around three million, and the city is administratively divided into 10 sub-cities and 99 Kebeles. In 2007, a total of 30 hospitals, 29 health centres and 442 clinics were providing health care services [12]. Of the 58 health facilities offering maternity care services, 81% were providing PMTCT services. In 2007 alone, over 70,000 pregnant women who were eligible for PMTCT services were living in the city. Over 80% of these pregnant women were attending antenatal care services, yet only 33.1% of the deliveries were attended by skilled professionals. Of the 24, 584 pregnant women who received HIV counselling and testing in 2007, 7.2% were HIV positive [12].

The first national PMTCT guidelines were developed in 2001 in preparation to launch a PMTCT programme [4]. In 2004, three years after the development of the first PMTCT guidelines, a free PMTCT programme was launched in selected public health facilities [4]. Initially, only six public health centres were offering PMTCT services across the city. The PMTCT programme continued to expand and from 2007 the programme has been scaled up to private facilities. As of April 2009, of a total of 52 facilities offered PMTCT services, 25 of them being private facilities (unpublished report from Addis Ababa City Administration Health Bureau). All the PMTCT facilities were integrated with Antenatal Care (ANC) service and the majority also provided delivery services.

For the monitoring and evaluation of HIV prevention programmes, a M&E system was established in 2003. As part of the M&E system, the monthly PMTCT reporting started in February 2004 with Addis Ketema sub-city. From July 2005, all the sub-cities reported. The PMTCT reporting format developed by the Ministry of Health includes output indicators. The format has been distributed to all service outlets by respective sub-city health bureaus. The PMTCT reports were first collected from the log books at the service outlets and then reported to the respective sub-cities by the end of each month. In the sub-cities, the reports from all private and public facilities were compiled and reported to Addis Ababa City Administration Health Bureau. In this study we collected the monthly PMTCT reports from the 10 sub-cities where they compiled data from all service outlets that started reporting at different times.

The first national PMTCT guidelines was revised in July 2007 to accommodate changes in policies made by the WHO from 2004 to 2006 [6]. Until the revised guidelines came into effect in early 2008, the HIV counselling and testing was offered as an opt-in approach [13], and sdNVP was given to positive women during pregnancy and to their infants within 72 hours of birth [14]. The HIV positive pregnant women were advised to feed exclusive formula if AFASS; if not, exclusive breast feeding [15].

According to the revised guidelines, pregnant women attending ANC should be offered HIV counselling and testing routinely as an opt-out approach [5,6]. The HIV positive pregnant women should start ZDV from 28 weeks of gestation plus intrapartum lamivudine and sdNVP followed by ZDV for seven days postpartum. For the infant, ZDV and sdNVP should be given at birth followed by ZDV for seven days if the mother received prophylaxis for at least one month, otherwise the infant should receive ZDV for one month postpartum [1,6,16]. The HIV positive pregnant women should receive infant feeding counselling in accordance with the 2006 WHO infant feeding update [6,7]. Moreover, HIV antigen testing of infants with polymerase chain reaction (PCR) at >= 45 days postpartum replaced the former antibody testing at >=18 months of age [6]. Nonetheless, early recommendations on partner testing, irrespective of the woman's HIV status, and referring HIV positive pregnant women for treatment, care and support remained unchanged with renewed emphasis in the revised guidelines [6].

### Data analysis

The reports were entered in Microsoft Excel spreadsheet and analyzed using Pivotal tables and charts. Prevalence and Relative risk (RR) were determined and χ^2 ^test for trend was analysed using the Epicalc 2000 software. The χ^2 ^trend tests were used to check whether there was a linear trend in PMTCT service utilization and HIV prevalence across the years and were reported as RR with associated p-values. The χ^2 ^tests for trend change for all outcome indicators were compared across the years taking 2004 as the reference time. P-value less than 0.05 was considered significant. Proportions are reported with 95% confidence intervals.

The following outcome indicators were analysed:

1) The proportion of women who received pre-test counselling, HIV testing and post-test counselling among new ANC attendees.

2) The proportion of women who tested positive and partners who received HIV testing among the total number of women who tested for HIV.

3) The proportion of women (and babies) who received ARV prophylaxis, infant feeding counselling and who were referred for treatment, care and support among women who tested positive for HIV.

4) The proportion of babies who tested positive among exposed babies tested for HIV.

### Ethical consideration

The study was reviewed and approved by the Regional Committee for Medical Research Ethics in Western Norway and the Ethical committee of Addis Ababa Region Health Bureau in Ethiopia. Study permit from Addis Ababa Region Health Bureau and respective sub-cities were obtained.

## Results

We obtained 565 PMTCT monthly reports from the 10 sub-cities. In Lideta sub-city three reports were missing and we collected them from the service outlets instead. In 6.5% (37) of the reports information on revisiting ANC attendees was either missing, or new and revisit attendees were not reported separately.

During February, 2004 to August, 2009, a total of 663 603 pregnant women attended ANC in health facilities offering PMTCT programme, and 24.6% (163 635) of them were new attendees. Overall, 135 986 women (new and revisit ANC attendees) and 4.9% (6 664) of their partners received HIV testing and 6.2% (8 467) of the women were HIV positive. Of the tested women, 97.1% (131 992) received post-test counselling. Among the HIV positive women, 42.4% (3 594) received ARV prophylaxis, 41.0% (3 474) were referred for treatment, care and support and 82.8% (7 010) received infant feeding counselling. Among babies born to the HIV positive women, 31.0% (2 621) received ARV prophylaxis and 10.6% (896) were tested for HIV (See Additional file 1: Compiled data from February, 2004 to August, 2009 from the 10 sub-cities).

### Trends in HIV counselling and testing

**Figure 1:**
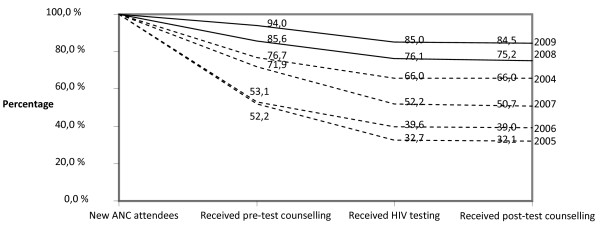
**Proportion of women who received pre-test counselling, testing and post-test counselling among new ANC attendees**. --- In 2004 to 2007 when the HIV testing was offered in an opt-in approach. ___In 2008 and 2009 when the HIV testing was offered routinely in an opt-out approach.

Fig 1 presents the year by year trend in HIV counselling and testing utilization. The proportion of women who received pre-test counselling, testing and post-test counselling among new ANC attendees was 66.3% (95% CI 64.8-67.7) in 2004 based on the reports from the three sub-cities that started reporting in 2004. In 2005, following the PMTCT programme scale up to the rest of the sub-cities, the counselling and testing utilization dropped to 32.1% (95% CI 31.5-32.7). Although the drop appeared to be marked in the three sub-cities that started reporting earlier, those that started later also showed a similar trend. The poor utilization persisted till 2007 when only 50.7% (95% CI 50.2-51.2) of the new attendees received HIV counselling and testing. The PMTCT programme gained momentum in 2008 when the revised guidelines that incorporated routine opt-out testing offer came into effect. Utilization of HIV counselling and testing increased to 84.5% (CI 84.1-84.9) in 2009. The trend in receiving post-test counselling remained stable at high level across the years which imply that almost all the tested women collected their HIV test result (Fig 1).

There were variations across the sub-cities in the proportion of women who had not received pre-test counselling or testing. In 2009, one year following the shift to routine opt-out approach, 12.0%, 18.4%, 19.3%, 14.0%, 14.6% and 27.3% of the women attending ANC in Addis Ketema, Arada, Bole, Kirkos, Lideta and Nifas Silk Lafto sub-city, respectively, did not receive pre-test counselling. Similarly, among women who received pre-test counselling in 2009, 17.3%, 11.3%, 17.0% and 24.8% in Arada, Bole, Gulele and Kirkos sub-cities, respectively, did not receive HIV testing.

### HIV testing versus HIV prevalence

**Figure 2:**
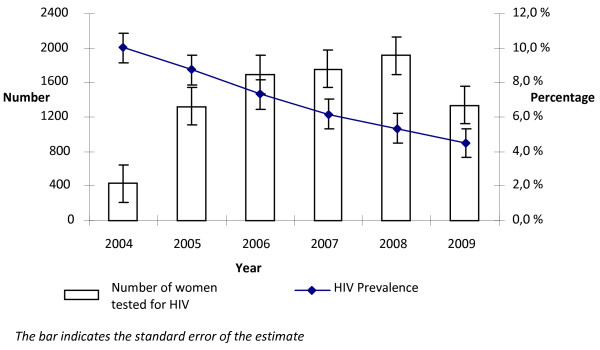
**Trends in HIV prevalence among antenatal care attendees tested in PMTCT settings from 2004 to 2009 in Addis Ababa**.

Fig 2 presents the overall trends in HIV prevalence among ANC attendees across the years. The HIV prevalence among the total ANC attendees (both new and revisit) who tested for HIV appeared to decline steadily from 10.5% (CI 9.6 -11.5) in 2004 to 4.6% (95% CI 4.3 - 4.8) in 2009 (RR 0.46) in parallel with the increasing number of women testing for HIV. During the same period the trends in HIV prevalence across the sub-cities showed significant decline, except Yeka sub-city where the HIV prevalence actually increased by 17% in 2009 compared to 2004 (RR 1.17, p < 0.05) (Table 1).

**Table 1:** HIV prevalence among antenatal care attendees who tested for HIV, relative risk and χ^2 ^for trend test across the years by sub-city

Sub-city	2004		2005		2006		2007		2008		2009		**χ**^**2 **^**trend**
		
	% (n)	RR	% (n)	RR	% (n)	RR	% (n)	RR	% (n)	RR	% (n)	RR	
**Addis Ketema**	12.8(195)	Ref	11.1(168)	0.99	7.4(170)	0.66	7.8(231)	0.70	4.6(178)	0.41	4.7(130)	0.42	127.3 *
**Akaki Kaliti**			8.7(99)	Ref	7.0(131)	0.81	6.8(16)	0.78	4.9(136)	0.57	5.3(113)	0.61	22.3 *
**Arada**			8.9(134)	Ref	8.2(185)	0.92	6.3(159)	0.71	5.9(298)	0.66	3.8(123)	0.43	64.4 *
**Bole**	8.6(89)	Ref	10.0(112)	1.17	6.8(149)	0.79	5.5(158)	0.64	4.8(171)	0.56	3.2(130)	0.37	105.4*
**Gulele**			6.7(77)	Ref	6.6(118)	0.98	5.7(156)	0.84	5.0(136)	0.75	4.5(93)	0.67	12.1**
**Kirkos**			10.2(89)	Ref	9.5(188)	0.93	7.1(162)	0.69	6.8(191)	0.66	4.6(122)	0.44	54.3 *
**Kolfe Keraniyo**			5.4(67)	Ref	4.9(75)	0.91	2.8(70)	0.51	3.3(73)	0.62	3.7(81)	0.69	7.7^‡^
**Lideta**	9.6(148)	Ref	10.2(244)	1.06	6.8(198)	0.71	5.9(228)	0.62	4.8(246)	0.50	4.6(217)	0.48	119.8*
**Nifas-silk Lafto**			8.0(175)	Ref	7.5(253)	0.93	6.0(232)	0.74	4.6(217)	0.57	4.0(140)	0.49	70.5*
**Yeka**			7.9(161)	Ref	7.6(228)	0.96	8.2(206)	1.03	9.3(268)	1.17	9.2(193)	1.17	6.2^‡^

Ref = Reference *P < 0.0001 **P < 0,001 ‡P < 0,01 ‡ ‡ P < 0,05 NB: The denominator for each cell is different

### ARV Prophylaxis uptake

**Figure 3:**
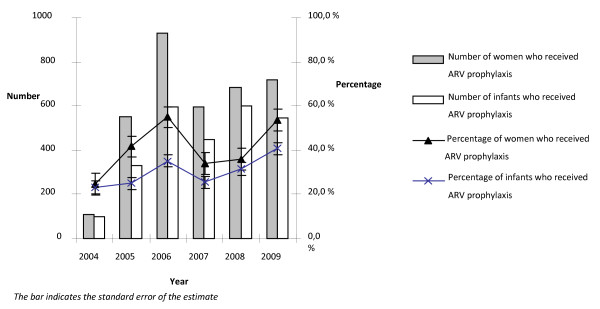
**Percentage of women and babies who received ARV prophylaxis from 2004 to 2009 in Addis Ababa**.

Of the 8 467 HIV positive women, ARV prophylaxis was given to 42.4% (95% CI 41.4-43.5) and to 31.0% (95% CI 30.0-31.0) of their infants. From early 2008, the prophylaxis regimen was shifted from sdNVP to multidrug ZDV regimen. Despite the big difference in the two regimens, the reports did not show marked changes of the trend in prophylactic ARV uptake. In 2004, the ARV prophylaxis uptake by the women and infants was low, but the gap in prophylaxis uptake between the women (24.8%. 95% CI 20.8-29.2) and babies (23.2%. 95% CI 19.3-27.5) was narrow. In 2005 and 2006 an increased proportion of women received prophylaxis, 41.6% (95% CI 39.0-44.3) and 55.0% (95% CI 52.3-57.4) respectively. In 2006 the proportion and number of women who received ARV prophylaxis peaked, yet only 35.1% (95% CI 32.8-37.4) of their infants received prophylaxis, resulting in the widest gap in prophylaxis uptake. In 2007, the prophylaxis uptake by the women and infants dropped to 33.8% (95% CI 31.6-36.1) and 25.4% (95% CI 23.4-27.5), respectively, and the gap narrowed again. The prophylaxis uptake shows an increasing trend since 2008 (Fig 3).

### Referral for treatment, care and support

The need for referring HIV positive pregnant women for treatment, care and support gained momentum in response to the 2004-2006 national roadmap to accelerate access to antiretroviral therapy [17] and the launching of the revised PMTCT guidelines [6]. The proportion of HIV positive pregnant women referred for treatment, care and support increased from 3.2% (95% CI 1.9-5.5) in 2004 to 59.9% (95% CI 57.2-62.5) in 2009. In other words, the HIV positive pregnant women in 2009 were over 18 times more likely to be referred for treatment, care and support than their counterparts in 2004 (RR 18.5 X^2 ^trend p < 0.0001).

### Infant feeding counselling

In 2004, when the PMTCT programme was first launched, 58.3% (95% CI 53.5-63.0) of the HIV positive women received infant feeding counselling, compared to 87.3% (95% CI 85.3-89.0) in 2009. The HIV positive women in 2009 had a 50% more chance to receive infant feeding counselling than the positive women in 2004 (RR 1.5, χ^2 ^trend p < 0.001). Despite the changes in infant feeding recommendation at different times, the report lack information on the kind of infant feeding advice given, infant feeding choice made by the women or pattern of infant feeding.

### HIV infection among exposed babies

**Figure 4:**
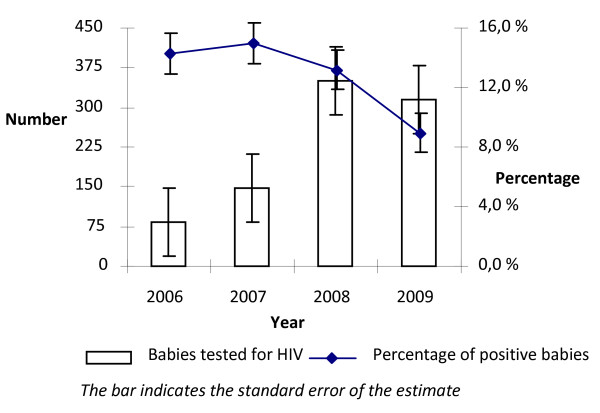
**HIV infection among exposed babies tested for HIV from 2006 to 2009 in Addis Ababa**.

Overall, only 10.6% (896) (95% CI 9.9-11.2) of the HIV positive women completed their follow up to child HIV testing. The rate of MTCT was evaluated based on the 896 babies tested for HIV during the period 2006-2009. Among the 896 exposed babies 106 were HIV positive. Of the babies on sdNVP regimen who tested at >=18 months with rapid antibody test, 14.3% (95% CI 7.92-24.0) in 2006 and 15.0% (95% CI 9.8-22.1) in 2007 were HIV positive. In 2009, among babies on the multidrug ZDV regimen who tested at >=45 days postpartum with antigen test using PCR, 8.2% (95% CI 5.55-11.97) were HIV positive (Fig 4).

### Partner testing

**Figure 5:**
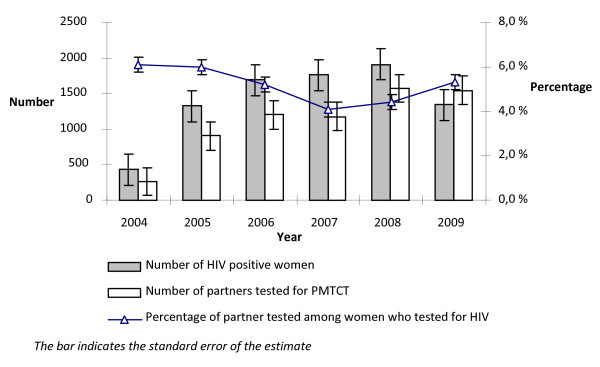
**Trends in partner testing in the PMTCT settings from 2004 to 2009 in Addis Ababa**.

The overall trend in the proportion of partners tested among women who received HIV testing remained stable at low level i.e 6.4% (95% CI 5.7-7.2) in 2004 and 5.3% (95% CI 5.0-5.5) in 2009. Compared to the women who were tested in 2004, women tested in 2009 were 14% less likely to be tested with their partner (RR 0.86, p < 0.01). However, there was an increase in the absolute number of partners tested across the years in parallel with the increased number of women who tested positive. In 2009 the number of partners tested was higher than the number of women who tested positive (Fig 5).

## Discussion

In this study we examined trends in PMTCT service utilization and assessed the rate of MTCT in Addis Ababa, Ethiopia. The HIV counselling and testing service utilization improved substantially in 2009 following policy shift to routine opt-out approach. The HIV prevalence appeared to decrease steadily paralleling the increased number of women tested for HIV. Irrespective of policy changes, the uptake of ARV prophylaxis and loss to follow up remained unimproved. Out of the 10.6% (896) exposed babies tested for HIV, the cumulative probability of HIV infection decreased to 15.0% by 2007 among babies on sdNVP regimen tested at >=18 months of age, and to 8.2% by 2009 among infants on ZDV regimen tested at >=45 days of age. The proportion of HIV positive pregnant women referred for treatment, care and support increased eighteen-fold in 2009 compared to 2004. Meanwhile, the proportion of partners tested in the PMTCT setting declined significantly by 14% from 2004 to 2009.

The proportion of women who received HIV counselling and testing among new ANC attendees increased significantly from 50.7% in 2007 to 84.5% in 2009 following the shift to routine opt-out testing. Consistent with our findings, studies from other resource poor settings have revealed significant improvement in HIV testing at ANC from 45% to 99% following the shift to routine opt-out approach [18-20]. Nevertheless, over 10% of the new ANC attendees had not received pre-test counselling or testing in 2009. In this regard the opt-out approach, which seems to be robust to maximise HIV testing in many settings [18-20], did not show remarkable success in our study. This could be attributed to two major reasons. First, most of the studies that reported nearly a 100% HIV test acceptance when testing was offered routinely as an opt-out approach, were pilot initiatives [18-20], unlike our study that compiled PMTCT reports from a national programme. In line with our argument, a study in Kenya based on data from 43 PMTCT service outlets reported a 80.6% HIV test acceptance [21]. Second, the expansion of the PMTCT programme to private facilities seem to have contributed to the persistence of a large proportion of women who had not received HIV counselling or testing even after the shift to routine testing. Site factors are reported to be more relevant than participant factors in determining HIV test acceptance in a study from Kenya [21]. In particular, characteristics of the provider appear to be an important determinant for HIV test acceptance. For instance, characteristics of the midwives were found to be an independent determinant for HIV testing uptake in England [22], whereas high test refusal was associated with HIV testing being offered by general practitioners in Canada [21,23]. In almost all the private facilities in Addis Ababa, ANC and HIV testing was offered by physicians who had little or no training on HIV counselling and testing in an opt-in approach. This indicates that there are gaps in the implementation of the routine opt-out testing strategy, particularly in private facilities. On top of that, the utilization of post-test counselling and collection of test result by almost all tested women throughout the years, irrespective of the testing approach employed, implies that the persistent gap in pre-test counselling and HIV testing service utilization could be an evidence of failure of the health system to deliver the programme rather than a failure of the women.

The HIV prevalence among the tested women had reduced by 54% in 2009 compared to the level in 2004, in parallel to the increased number of women being tested. Similar declining trend in HIV prevalence has been observed among adults and from a sentinel surveillance report in Ethiopia. Synergy between the natural progression of the HIV epidemic and behaviour changes among the general population in terms of increased condom use and reduced risky sexual behaviour seem to contribute to the declining prevalence [3,4]. The HIV prevalence estimate in our study was lower than that of the ANC sentinel surveillance report, i.e 6.2% vs 9.3% in 2007/2008 [12]. Consistent with our finding, a study that compiled data from Kenya, Ethiopia, and Zimbabwe for 2005 reported that the HIV prevalence in Ethiopia was 6.4% from PMTCT programme reports and 8.2% from sentinel surveillance reports [24-26]. The two reports, although generated from the same population, have differences in population size and timing of data collection [24-26]. The PMTCT report includes all women who participated in the PMTCT programme all year round. In the sentinel surveillance, leftover blood samples were collected from a smaller number of ANC attendees from selected facilities, often for a period of 3 months biennially.

The decreasing trend in the HIV prevalence estimate across the years could also be attributed to a more representative sample of pregnant women tested for HIV in our study. In other words, the inclusion of more service outlets and therefore reaching out to a large population in our study seems to be more representative, unlike the sentinel surveillance report that relied on reports from a few public facilities. Even in the sentinel surveillance reports, as the number of sentinel sites increased the estimated HIV prevalence declined [4]. More importantly we included reports from private facilities where the HIV prevalence is reported to be lower compared to ANC attendees in public facilities, 2.4% (unpublished report from Addis Ababa City Administration Health Bureau) vs 9.3% [12] respectively in 2007/2008. Moreover, the HIV prevention potential of Highly Active Antiretroviral Therapy (HAART) should not be ignored [27].

Despite the marked increase in HIV testing following the shift to routine opt-out approach, neither the proportion nor the number of women receiving ARV prophylaxis have increased. In 2009, only 53.7% of the women and 40.7% of their babies received ARV prophylaxis. Consistent with our findings, in Addis Ababa, 49.3% of the HIV positive women and 35.3% of their babies received ARV prophylaxis in 2007/2008 [28] and the programme achieved only 30% compared to 80% as the target [12]. Actually, poor ARV prophylaxis uptake is not a new story, even those intervention studies that show almost 100% successes in HIV testing were in short of ARV prophylaxis uptake [18-20]. However, the lack of improvement in ARV prophylaxis utilization following the shift to routine testing is an issue of great concern as the shift to an opt-out approach was primarily intended to increase the proportion of women and infants receiving prophylactic ARV drugs [5]. In the era of routine testing for HIV, the PMTCT programme appears to be an effective screening programme than a prevention programme due to the large number of dropouts after testing. Yet, the gain in HIV testing turnout limits the weakness of the routine opt-out approach in terms of subsequent dropouts and poor adherence to ARV prophylaxis [29]. Currently the availability of potent ARV prophylaxis is changing the landscape, and even mother-to-child HIV transmission through breast feeding has become less of a concern [30]. Yet, still more lives are at stake because of the lack of improvement in uptake of this critical component of the PMTCT programme.

According to Kasenga et al., skilled attendance at birth is an important determinant of ARV prophylaxis uptake that requires thorough consideration [31]. In Addis Ababa, only 30% of the pregnant women had skilled attendance at birth in 2008 [12] corresponding to the proportion of infants who received ARV prophylaxis [28]. Since infants are given prophylaxis within 72 hours of birth at the health facility, infants delivered at home have less chance to receive ARV prophylaxis than infants delivered in health facilities. An intervention study from Zambia gives some hope that dropouts and non-adherence to ARV prophylaxis could be reduced to zero. This study, which employed multiple interventions, increased the ARV prophylaxis uptake from 29% at baseline to 100% within 3 years [32]. This gives reassurance that the official 80% ARV prophylaxis uptake goal for Ethiopia could be achieved through concerted effort and renewed commitment.

According to the findings, an increasing proportion of HIV-positive pregnant women were referred for treatment, care and support services, from 3.2% in 2004 to 59.9% in 2009. The referral is intended for prompt initiation of ARV prophylaxis or treatment for eligible women using CD_4 _or lymphocyte count and WHO staging criteria [6]. By doing so, the programme is addressing the most important issues that PMTCT programmes have been criticized for, i.e the lack of sensitivity to the needs of pregnant women. The PMTCT programme is increasingly successful in bridging the gap between prevention and treatment to address the moral and ethical questions raised over the years [1]. The eighteen-fold increase in the proportion of positive pregnant women being referred for treatment, care and support from 2004 to 2009 demonstrates the good will and the potential to integrate new policies and strategies. Yet, more has to be done to ensure that all HIV-positive pregnant women have access to prophylaxis or treatment, care and support.

The ultimate objective of a PMTCT programme is to avert new HIV infections among children. However, only 10.6% (896) of the HIV positive pregnant women completed their follow up to infant HIV testing. The rates of MTCT were therefore assessed based on test result of the 896 babies tested for HIV. In the absence of any PMTCT intervention the cumulative probability of HIV infection among exposed babies aged >=18 months is in the range of 29% to 47% according to a cohort study conducted in an orphanage in Addis Ababa [4]. In our study, the cumulative probability of HIV infection among exposed babies on sdNVP regimen tested at >=18 months was 14.3% in 2006 and 14.9% in 2007. According to the HIVNET 012 randomized trial, sdNVP regimen has a 41% efficacy. In this trial, consistent with our findings, the cumulative probability of infant HIV infection is 15.7% among breast fed infants tested at >=18 months [14]. However, a methodologically similar study from Malawi that compiled monthly reports showed a 15.5% HIV infection rate among infants on sdNVP regimen tested at 6 weeks postpartum without accounting for the breast feeding transmission [33]. Nevertheless, there is a possibility that our estimate could be biased due to the large loss to follow up.

In 2009, 8.2% of the exposed infants on ZDV regimen tested at 45 days were HIV positive. In the Petra clinical trial, multidrug ZDV regimen showed 63% efficacy in reducing MTCT. In this trial the rate of HIV transmission among infants on multidrug ZDV regimen tested at 45 days was 5.7% [16]. Considering the fact that our data are generated from a national PMTCT programme and the obvious methodological difference with the Petra trial, the 8.2% infant infection rate reported in our study indicates the success of the national PMTCT programme among those who completed their follow up to infant HIV testing. A cohort study from similar resource poor settings that evaluated the effectiveness of a PMTCT programme among predominantly formula fed infants on ZDV regimen tested at >=45 days reported a 9.1% cumulative infant HIV infection, higher than the rate of HIV infection reported in our study [34]. However, since the HIV testing was done at >=45 days, those HIV negative infants who continue to breast feed are still at risk of acquiring new infection. In general, the rate of MTCT averted by the national PMTCT programme appears promising among those who adhered to the programme. Nevertheless, the possibility of underestimation cannot be excluded since we lack information on loss to follow up. In line with this limitation, Ahoua et al. found that the cumulative probability of infant HIV infection among tested infants was 8.3%, whereas it was 15.5% when HIV related deaths were included in the analysis [35].

The last, but not least important PMTCT programme outcome indicator examined in our study was partner testing. The proportion of partners tested remained very low with a 14% significant decline in 2009 compared to 2004. A review on couple centred counselling shows that partner involvement in HIV testing not only helps to increase disclosure, condom use and uptake of ARV prophylaxis but also contributes to the lower rate of seroconversion compared to individual counselling [8]. We noted a parallel trend in the number of partners tested with the number of women who tested positive across the years. This indicates that most of the partners who came for HIV testing were those whose wives tested positive. Partner testing in the context of PMTCT seems to facilitate women's coping, yet missing out the important prevention aspect by not advising HIV negative women to bring their partner for testing.

A study from South Africa shows that 3% of the pregnant women who were found to be HIV negative in their first HIV testing during pregnancy became HIV positive in repeat test in late pregnancy, giving a 10.7% incidence per year [36]. This indicates that women are at risk to acquire new HIV infection from their HIV positive partner anytime during pregnancy and even during breast feeding. In eastern and southern African region, including Ethiopia, 36-85% of HIV positive individuals are believed to live with an HIV negative partner [4,10]. Discordant couples are the newly identified high risk group in Ethiopia, as most infections are occurring within marriage. Because of mucosal and hormonal changes during pregnancy, the HIV incidence is four times higher among pregnant women compared to their non-pregnant counterparts [37]. Meanwhile, women having recent HIV infection are more likely to transmit HIV infection to their babies [36]. Therefore, it is crucial to focus on partner testing and involvement in the PMTCT programme to optimise programme effectiveness. The current strategies in Addis Ababa, that include giving priority for women coming with their partner for testing and sending an invitation home to partner should be encouraged.

One of the limitations of our study is that the rate of MTCT was examined based on infants tested for HIV, which could actually be underestimated due to the large loss to follow up. Considering the lack of a system to trace loss to follow up, our finding still highlights the potentially averted infections. Another limitation is that the PMTCT programme reached out to only 80% of pregnant women due to incomplete ANC attendance [12] and the findings seem not to represent the whole nation, where a high proportion of the population is rural. However, since it was generated from a national programme, lessons learnt herein could benefit the PMTCT programme across the country. We also believe that our findings are generalisable to the big cities where the HIV prevalence is higher.

Retrospective data collected primarily for reporting purposes always have weakness, especially in resource poor settings where the quality of reports are often questionable [11]. By limiting our study objectives to those indicators that could be calculated from the reports we minimized the risk of having incomplete data. We obtained almost all the reports from February 2004 to August 2009 from the 10 sub-cities. To validate our data we checked Addis Ababa City Administration Health Bureau and NGO reports, and our data were found to be consistent. For missing reports we obtained data from log books and reports at service outlets.

## Conclusion

Our findings suggest that the proportion of women receiving HIV counselling and testing has increased substantially from 2004 to 2009. The shift in HIV testing approach has further reduced the proportion of women not receiving HIV counselling and testing. However, the lack of improvement in ARV prophylaxis uptake after the shift to routine opt-out testing is an issue of great concern as the shift was intended to facilitate access to ARV prophylaxis. The health system appeared not only to fail to deliver the most critical component of the PMTCT programme, i.e ARV prophylaxis, but also to retain the HIV positive women to complete their follow up. The effectiveness of the national PMTCT programme is questionable as only 10% of the HIV positive women completed their follow up to infant HIV testing. A tracing system for loss to follow up should be in place for measuring the actual impact of the programme. In addition, the sub-cities and the Addis Ababa city Administration Health Bureau must devise a mechanism to increase prophylaxis uptake and to retain the HIV positive women in the programme until the follow up is completed. The counselling Training should emphasize partner testing to ensure that all women tested in PMTCT settings receive counselling on partner testing, irrespective of their HIV status. Further research is imperative to identify and address challenges to ARV prophylaxis uptake, partner testing, referral for treatment, care and support and challenges to follow up. We recommend the health facilities, sub-cities and the City Administration Health Bureaus to utilize the monthly reports to identify gaps in service utilization and to come up with context specific solutions for the realisation of the country's goal of "HIV free generation by 2020".

## Competing interests

The authors declare that they have no competing interests.

## Authors' contributions

AHM prepared the study proposal, collected and analyzed the data, interpreted the findings and wrote the manuscript. OM was involved in developing the study proposal, supervising the data collection and revising the manuscript. SGH was involved in developing the study proposal and revising the manuscript. All authors have read and approved the final manuscript.

## Pre-publication history

The pre-publication history for this paper can be accessed here:

http://www.biomedcentral.com/1472-6963/10/267/prepub

## Supplementary Material

Additional file 1 **Compiled monthly PMTCT reports from February, 2004 to August, 2009 for the 10 Sub-cities in Addis Ababa**. The table containes monthly PMTCT reports from February, 2004 to August 2009 collected from the 10 sub-cities. Addis Ketema, Bole and Lideta sub-cities started reporting from 2004 while the rest of the sub-cities reported from 2005. Click here for file
