# Supplementary material for: Promising outcomes of a national programme for the prevention of mother-to-child HIV transmission in Addis Ababa: a retrospective study
Source: BMC Health Serv Res. 2010 Sep 9;10:267. doi: 10.1186/1472-6963-10-267 (PMC2944274; doi:10.1186/1472-6963-10-267)
Supplement: Additional file 1 — Compiled monthly PMTCT reports from February, 2004 to August, 2009 for the 10 Sub-cities in Addis Ababa. The table containes monthly PMTCT reports from February, 2004 to August 2009 collected from the 10 sub-cities. Addis Ketema, Bole and Lideta sub-cities started reporting from 2004 while the rest of the sub-cities reported from 2005. [file 1472-6963-10-267-S1.PDF]

Variable	Sub-city										
	Addis Ketema	Bole	Lideta	Akaki Kaliti	Arada	Gulele	Nifas-silk Lafto	Yeka	Kirkos	Kolfe Keraniyo	Total
ANC attendees (N)*	15 971	18 276	24 168	11 625	22 130	12 906	21 544	12 806	13 061	11 148	163 635
ANC attendees (R)**	40 183	45 109	70 468	39 988	72 400	33 125	79 420	42 033	41 120	32 591	494 437
ANC attendees (T)***	56 866	63 385	97 340	50 297	94 530	46 031	100 964	55 236	55 133	43 821	663 603
Pre-test counselled (N)	12 753	11 414	19 642	7 403	16 013	10 038	11 170	12 106	9 079	8 224	117 842
Pre-test counselled (R)	2 097	4 896	27 907	736	17 514	3 034	8 647	9 147	8 797	5 725	91 500
Pre-test counselled (T)	15 232	16 487	50 304	11 139	33 527	13 072	19 917	21 253	18 730	13 939	213 026
Received HIV testing (N)	12 485	10 493	13 737	7 269	11 110	8 707	10 278	8 019	6 416	6 533	95 056
Received HIV testing (R)	2 049	4 164	5 747	2 966	3 452	1 706	7 385	3 782	3 588	3 122	37 961
Received HIV testing (T)	14 895	14 827	20 550	10 235	14 562	10 413	17 672	12 558	10 619	9 655	135 986
Post-test counselled (N)	12 470	10 277	13 568	7 180	10 865	8 623	10 238	7 863	6 379	6 350	93 791
Post- test counselled (R)	2 046	4 052	5 552	2 904	3 103	1 690	7 263	3 764	3 583	2 653	36 610
Post- test counselled (T)	14 877	14 499	20 162	10 084	13 968	10 313	17 501	11 187	10 398	9 003	131 992
Tested positive	1072	809	1 281	635	899	580	1 017	1 056	752	366	8 467
Women who received ARV prophylaxis	634	272	440	309	379	249	510	399	253	149	3 594
Babies who received ARV prophylaxis	492	283	292	228	279	161	377	238	180	91	2 621
Women who received infant feeding counselling	921	508	1 153	540	776	548	930	670	608	356	7 010
Women who referred for treatment, care & support	449	314	548	308	351	297	285	318	432	172	3 474
Babies tested for HIV	256	76	117	101	42	68	100	21	78	37	896
Partner tested for PMTCT	817	563	842	440	1133	681	912	630	294	352	6 664

** New ** Revisit ***Total*
